# Influence of the gel thickness on *in vivo* hyaline cartilage regeneration induced by double-network gel implanted at the bottom of a large osteochondral defect: Short-term results

**DOI:** 10.1186/1471-2474-14-50

**Published:** 2013-01-31

**Authors:** Hidetoshi Matsuda, Nobuto Kitamura, Takayuki Kurokawa, Kazunobu Arakaki, Jian Ping Gong, Fuminori Kanaya, Kazunori Yasuda

**Affiliations:** 1 Department of Sports Medicine and Joint Surgery, Graduate School of Medicine, Hokkaido University, Sapporo, Japan; 2 Department of Orthopedic Surgery, Faculty of Medicine, University of the Ryukyus, Okinawa, Japan; 3 Laboratory of Soft and Wet Matter, Department of Advanced Transdisciplinary Sciences, Faculty of Advanced Life Science, Hokkaido University, Sapporo, Japan

**Keywords:** Hyaline cartilage, Cartilage repair, Double-network hydrogel, Polymer

## Abstract

**Background:**

A double-network (DN) gel, which is composed of poly(2-acrylamido-2-methylpropanesulfonic acid) and poly(N,N’-dimethyl acrylamide), can induce hyaline cartilage regeneration *in vivo* in a large osteochondral defect. The purpose of this study was to clarify the influence of the thickness of the implanted DN gel on the induction ability of hyaline cartilage regeneration.

**Methods:**

Thirty-eight mature rabbits were used in this study. We created an osteochondral defect having a diameter of 4.3-mm in the patellofemoral joint. The knees were randomly divided into 4 groups (Group I: 0.5-mm thick gel, Group II: 1.0-mm thick gel, Group III: 5.0-mm thick gel, and Group IV: untreated control). Animals in each group were further divided into 3 sub-groups depending on the gel implant position (2.0-, 3.0-, or 4.0-mm depth from the articular surface) in the defect. The regenerated tissues were evaluated with the Wayne’s gross and histological grading scales and real time PCR analysis of the cartilage marker genes at 4 weeks.

**Results:**

According to the total Wayne’s score, when the depth of the final vacant space was set at 2.0 mm, the scores in Groups I, II, and III were significantly greater than that Group IV (p < 0.05), although there were no significant differences between Groups I and IV at a 3.0-mm deep vacant space. The expression levels of type-2 collagen in Groups II and III were significantly higher (p < 0.05) than that in Group IV.

**Conclusions:**

The 1.0-mm thick DN gel sheet had the same ability to induce hyaline cartilage regeneration as the 5.0-mm thick DN gel plug. However, the induction ability of the 0.5-mm thick sheet was significantly lower when compared with the 1.0-mm thick gel sheet. The 1.0-mm DN gel sheet is a promising device to establish a cell-free cartilage regeneration strategy that minimizes bone loss from the gel implantation.

## Background

Cartilage defects are common in symptomatic knees and an increasing health care concern. It has been a common belief that the articular hyaline cartilage tissue cannot spontaneously regenerate *in vivo*[1,2]. Therefore, the most prevalent strategy to repair the osteochondral defect is to fill the defect with a tissue-engineered cartilage-like tissue or a cell-seeded scaffold material [3-10]. Recently, some investigators have tried to fill an osteochondral defect with acellular polymer scaffolds to induce cartilage cell regeneration [11-14]. However, it could not be applied for clinical use due to various hurdles. Thus, functional repair of articular cartilage defects remains a major challenge in tissue regeneration medicine.

Recently, we have found that, when we implant a 5 to 8-mm thick PAMPS/PDMAAm double-network (DN) gel plug at the bottom of a large osteochondral defect created in the rabbit knee joint so that a 2- to 3-mm deep vacant space is intentionally left in the defect, cartilage regeneration can be induced *in vivo* within only 4 weeks in the vacant space [15]. This DN gel is composed of poly (2-acrylamido-2-methylpropanesulfonic acid) (PAMPS) and poly (N,N´-dimethyl acrylamide) (PDMAAm). This discovery has proposed a novel strategy to repair an osteochondral defect in the field of joint surgery using the artificially synthesized hydrogel without any cultured cells or mammalian-derived scaffolds. We, therefore, have confirmed the efficacy of this therapeutic strategy with the 5-mm thick DN gel plug not only in the patellofemoral joint but also in the femorotibial joint [16,17]. However, this therapeutic strategy with use of the 5-mm thick DN gel plug has some drawbacks from the clinical viewpoint: Namely, to implant the 5-mm thick DN gel plug, we have to make a 7- to 8-mm deep bone defect by surgically scraping the bottom of the defect. After the cartilage regeneration will be achieved, the DN gel plug will remain unabsorbed in the bone. Such intra-osseous existence of the 5-mm thick DN gel plug may not be adverse; however, it may become a future problem in the case of revision surgery due to various reasons such as infection, accidental trauma, and knee arthroplasty. The thinner DN gel implant is better from the clinical viewpoint in order to reduce the bone loss caused by the DN gel implantation. However, there is a possibility that a thin DN gel sheet may not have the same ability as the 5-mm thick gel plug concerning induction of the *in vivo* cartilage regeneration, because it may affect biomechanical environment in the defect. Thus, we have conducted this *in vivo* study in order to clarify if the gel thickness influences on the in vivo hyaline cartilage regeneration induced by the DN gel implanted at the bottom of a large osteochondral defect.

Recently, we have developed a DN gel sheet thinner than 1.0 mm with our advanced gel technology. In this study, we have hypothesized that a 1-mm thin PAMPS/PDMAAm DN gel sheet may be able to induce cartilage regeneration in an osteochondral defect, when it was implanted at the bottom of the defect so that a 2- to 3-mm deep vacant space was left in the defect, but that a 0.5-mm thin DN gel sheet may not be able to induce cartilage regeneration. The specific aim of this study was to test this hypothesis.

## Methods

### Materials

The PAMPS/PDMAAm DN gel is a kind of interpenetrating network hydrogel, but with an asymmetric structure: In the DN gel, the two independently cross-linked polymer networks are physically entangled with each other. This DN gel is strong enough (the compressive fracture strength of 3.1 MPa [18]) to create an implant plug, although the single network PAMPS and PDMAAm gels are too fragile as an implant in our pilot study. The elastic modulus of the PAMPS/PDMAAm DN gel is 0.20 MPa [18].

The PAMPS/PDMAAm DN gel was synthesized using the previously reported two-step sequential polymerization method [19]. Briefly, PAMPS hydrogel was obtained by radical polymerization using N,N^’^-methylenebisacrylamide (MBAA) as a cross-linker and 2-oxoglutaric acid as an initiator. The monomer concentration was 1 mol/l for PAMPS, 4 mol% for the cross-linker, and 0.1 mol% for the initiator. Aqueous solution containing a monomer, cross-linker, and the initiator was bubbled with nitrogen for 30 minutes, and then injected into a cell consisting of a pair of glass plates separated by a silicone rubber. The cell was irradiated with a UV lamp (wave length 365 nm) for about 6 hours. The DN gel was synthesized by the sequential network formation technique (two-step method). The PAMPS hydrogel (1^st^ network) was immersed in an aqueous solution of 2 mol/L DMAAm, containing 0.1 mol% MBAA, and 0.1 mol% 2-oxoglutaric acid for one day until reaching the equilibrium. The 2^nd^ network (PDMAAm) was subsequently polymerized in the presence of the PAMPS hydrogel by irradiating UV for 6 hours between two plates of glasses. After polymerization, the PAMPS/PDMAAm DN gel was immersed in pure water for 1 week and the water was changed twice daily to remove any un-reacted materials. Using this principle method, we created 5.0-mm thick DN gel blocks and 1.0- and 0.5-mm thick DN gel sheets, and then, 4.5-mm diameter plugs or sheets were punched out from each block or sheet. Subsequently, we prepared 5.0-mm thick DN gel plugs as well as 1.0- and 0.5-mm thick DN gel sheets, all of which had a 4.5-mm diameter.

### Study design and animal experimentation

A total of 38 mature female Japanese white rabbits, 6 months old and weighing 3.6 ± 0.7 kg, were used in this study. Animal experiments were carried out in the Institute of Animal Experimentation, Hokkaido University School of Medicine under the Rules and Regulation of the Animal Care and Use Committee, Hokkaido University School of Medicine (Reference number: 08-0068).

**Figure 1:**
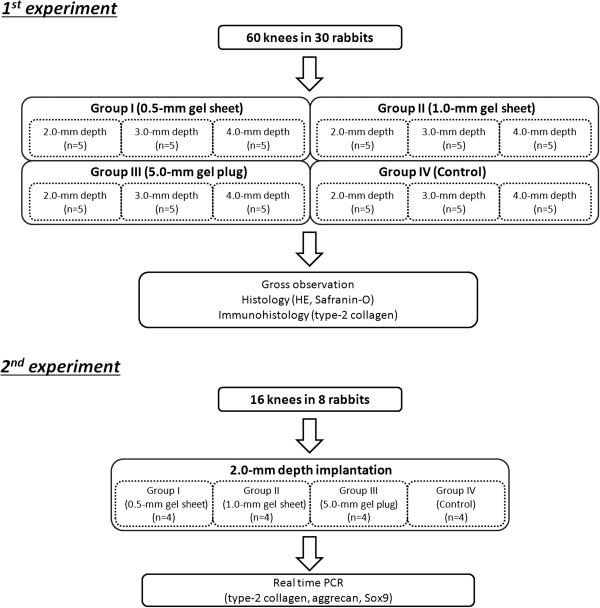
**Study design flowchart.** In the first study, sixty knees of the 30 rabbits were randomly divided into 4 groups of 15 knees each in order to clarify the effect of gel implant position (depth from the articular surface) in the defect on quality of the regenerated cartilage. The second study was conducted using 8 rabbits to evaluate gene expression of type-2 collagen, aggrecan, and Sox9 in the tissue regenerated in the defect using real-time PCR analysis.

**Figure 2:**
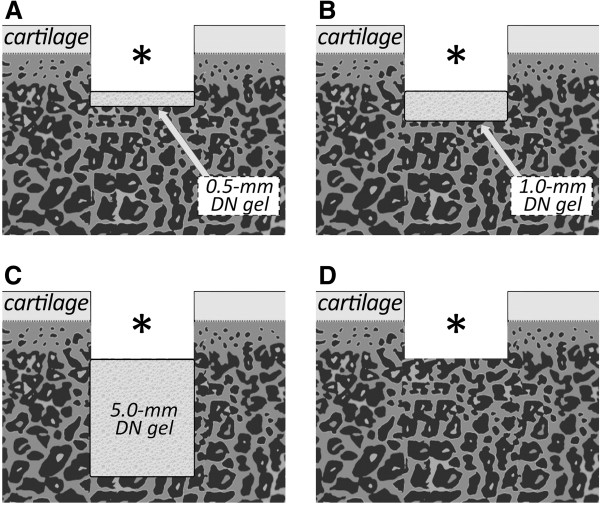
**A schematic example of implantation of DN gel plugs and sheets.A**; a 0.5-mm gel sheet was implanted at the bottom of the defect. **B**; a 1.0-mm gel sheet was implanted at the bottom of the defect. **C**; a 5.0-mm gel plug was implanted at the bottom of the defect. **D**; the control group has a defect left untreated. In each subgroup, the depth of the final vacant space in the defect with or without DN gel implantation became 2.0, 3.0, or 4.0 mm. For example, when the depth of the final vacant space was set at 2.0 mm in each Group, a defect was created with a depth of 2.5 mm in Group I, 3.0 mm in Group II, 7.0 mm in Group III, and 2.0 mm in Group IV using drill, and then the DN gel sheet or plug was implanted into the defect so that a vacant space having a 2-mm depth from the articular cartilage surface remained after surgery. Note that a vacant space (_*_) having the same depth from the articular cartilage surface remained after surgery in the same sub-group.

In the first experiment, we used 30 rabbits. The bilateral 60 knees of the 30 rabbits were randomly divided into 4 groups of 15 knees each (Figure 1). An operation for each animal was performed under intravenous anesthesia (pentobarbital, 25 mg/kg) and sterile conditions. In each knee, we created an osteochondral defect having a diameter of 4.3-mm in the femoral groove of the patellofemoral joint using the previously reported procedure [15,16]. In Group I, we implanted a 0.5-mm thick DN gel sheet at the bottom of the osteochondral defect. In Group II, we implanted a 1.0-mm thick DN gel sheet at the bottom of the osteochondral defect. In Group III, we implanted a 5.0-mm thick DN gel plug in the same manner reported in our previous study. In Group IV, a defect was left vacant without any implantation (Figure 2). In each group, the 15 knees were divided into 3 subgroups of 5 knees each. In each subgroup, the defect was created so that the depth of the final vacant space in the defect with or without DN gel implantation became 2.0, 3.0, or 4.0 mm, because our previous study showed that the regeneration effect of DN gel implantation was observed in this range of the vacant space depth [15]. For example, when the depth of the final vacant space was set at 2.0 mm in each Group, a defect was created with a depth of 2.5 mm in Group I, 3.0 mm in Group II, 7.0 mm in Group III, and 2.0 mm in Group IV using drill, and then the DN gel sheet or plug was implanted into the defect so that a vacant space having a 2-mm depth from the articular cartilage surface remained after surgery. Then, the incised joint capsule and the skin wound were closed in layers with 3–0 nylon sutures, and an antiseptic spray dressing was applied. Postoperatively, each animal was allowed unrestricted activity in a cage (310 x 550 x 320 mm) without any joint immobilization. All rabbits were sacrificed at 4 weeks and used for histological and immunohistochemical evaluations after gross observations.

The second experiment was conducted using the remaining 8 rabbits, based on the first study results. The 16 knees in 8 rabbits were randomly divided into 4 groups same manner as made in the first experiment and the final vacant space was set at 2.0-mm depth. All rabbits were sacrificed at 4 weeks, and real time polymerase chain reaction (PCR) analysis was performed (Figure 1).

### Examination methods

#### Gross observation for in vivo regenerated tissues

Immediately after sacrifice, the tissue regenerated in the osteochondral defect was quantitatively evaluated with the grading scale reported by Wayne et al. [14] Gross appearance of each defect on the femoral condyle was graded as to coverage (4 points), tissue color (4 points), defect margins (4 points), and surface (4 points). Thus, the maximum total score was 16 points.

#### Histological and immunohistochemical examinations

A distal portion of the resected femur was fixed in a 10% neutral buffered formalin solution for 3 days, decalcified with 50 mM EDTA for a period of 3–4 weeks, and then cast in a paraffin block. The femur was sectioned perpendicular to the longitudinal axis, and stained with hematoxylin-eosin and Safranin-O. For immunohistochemical evaluations, monoclonal antibody (anti-hCL (II), purified IgG, Fuji Chemical Industries Ltd, Toyama, Japan) was used as primary antibodies. Immunostaining was carried out according to the manufacturer’s instructions using the Envision immunostaining system (DAKO Japan, Kyoto, Japan). Finally, the sections were counterstained with hematoxylin. Histology was evaluated with the scoring system reported by Wayne et al. [14], which was composed of matrix points (4 points), cell distribution points (3 points), smoothness points of the surface (4 points), Safranin O stain points (4 points), Safranin O-stained area points (4 points). Thus, the maximum total score was 19 points.

#### Real time PCR analysis

Total RNA was extracted from the tissues regenerated in the defect, using the RNeasy mini kit (Qiagen Inc., Valencia, CA). RNA quality from each sample was assured by the A260/280 absorbance ratio. The RNA (100 ng) was reverse-transcribed into single strand cDNA using PrimeScript® RT reagent Kit (TakaraBio, Ohtsu, Japan). The RT reaction was carried out for 15 minutes at 37 degrees Celsius and then for 5 seconds at 85 degrees Celsius. All oligonucleotide primer sets were designed based upon the published mRNA sequence. The expected amplicon lengths ranged from 93 to 189 bp. The sequences of primers used in real time PCR analyses for rabbit regenerative tissues were as follows: type-2 collagen forward GACCATCAATGGCGGCTTC; reverse CACGCTGTTCTTGCAGTGGTAG. Aggrecan forward GCTACGACGCCATCTGCTAC; reverse GTCTGGACCGTGATGTCCTC. Sox9 forward AACGCCGAGCTCAGCAAGA; reverse TGGTACTTGTAGTCCGGGTGGTC. GAPDH forward CCCTCAATGACCACTTTGTGAA; reverse AGGCCATGTGGACCATGAG. The real time PCR was performed in Thermal Cycler Dice® TP800 (TakaraBio, Ohtsu, Japan) by using SYBR® Premix Ex TaqTM (TakaraBio, Ohtsu, Japan). cDNA template (5 ng) was used for real time PCR in a final volume of 25 microliter. cDNA was amplified according to the following condition: 95 degrees Celsius for 5 sec and 60 degrees Celsius for 30 sec at 40 amplification cycles. Fluorescence changes were monitored with SYBR Green after every cycle. A dissociation curve analysis was performed (0.5 degrees Celsius /sec increase from 60 to 95 degrees Celsius with continuous fluorescence readings) at the end of cycles to ensure that single PCR products were obtained. The amplicon size and reaction specificity were confirmed by 2.5% agarose gel electrophoresis. The results were evaluated using the Thermal Cycler Dice® Real Time System software program (TakaraBio, Ohtsu, Japan). Glyceroaldehyde-3-phosphate dehydrogenase (GAPDH) primers were used to normalize samples.

#### Statistical analysis

All data were described as the mean and standard deviation values. A commercially available software program (StatView 5.0, SAS Institute Inc., Cary, NC, USA) was used for statistical calculation. The mean value of the Wayne’s scores was statistically compared among groups using two-way analysis of variance (ANOVA). Regarding the gene expression of cartilage markers, one-way ANOVA was performed for each gene. The Fisher’s protected least significance difference test was used for post hoc multiple comparisons. The significance level was set at p = 0.05.

## Results

### Gross observation of the joint surface repair

**Figure 3:**
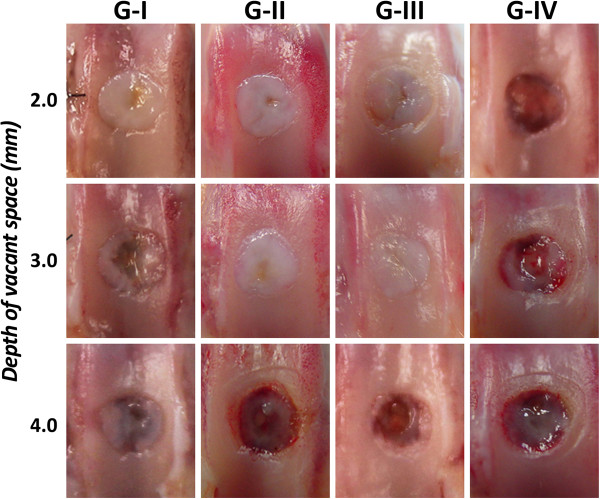
**Gross observations of the joint surface repair at 4 weeks.** The defect having 2.0-mm depth in G-I, II, and III was completely filled with a white opaque tissue. The defect having 3.0-mm depth in G- II and III was completely filled with a white opaque tissue, while the defect in G-I was filled with inhomogenous white and reddish tissue. The defect in G-IV showed reddish opaque, patchy tissues, independent of the depth. G-I; a 0.5-mm gel sheet was implanted at the bottom of the defect. G-II; a 1.0-mm gel sheet was implanted at the bottom of the defect. G-III; a 5.0-mm gel plug was implanted at the bottom of the defect. G-IV; the control group has a defect left untreated.

In gross observation of the joint surface repair, the defect having 2.0-mm depth with the 0.5-mm (Group-I), 1.0-mm (Group-II), or 5.0-mm (Group-III) thick DN gel implantation was completely filled with a white opaque tissue, while the untreated defect (Group-IV: control) surface showed reddish opaque, patchy tissues (Figure 3). The defect having 3.0-mm depth in Group-II, or -III was completely filled with a white opaque tissue, while the Group-I and Group-IV surface showed white and reddish opaque, patchy tissues (Figure 3).

### Histological and immunohistochemical evaluations

**Figure 4:**
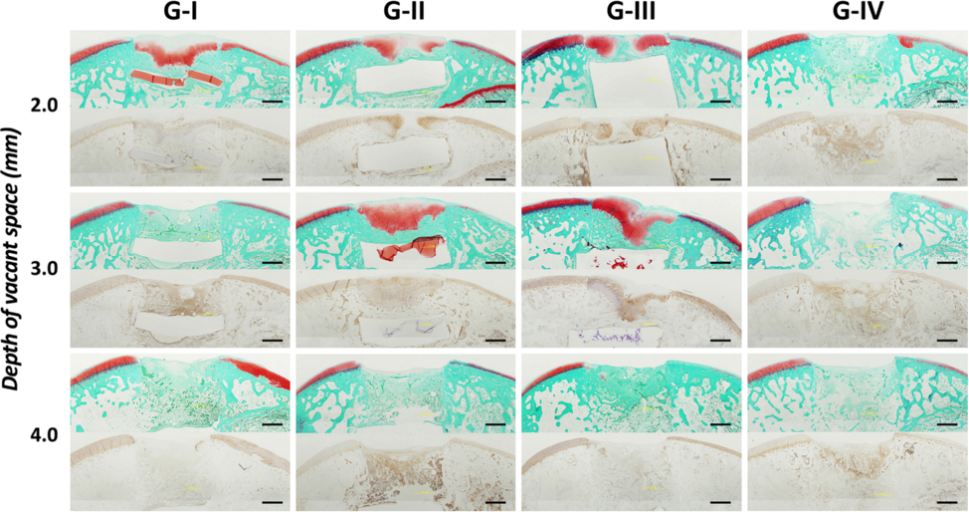
**Safranin-O and anti-type-2 collagen immunohistochemical staining at 4 weeks (2x original magnification).** The defect in G-I, II, and III was filled with a sufficient volume of the proteoglycan-rich tissue with regenerated subchondral bone tissue when the final vacant space was set at 2.0 mm. The DN gel implantation in G-II and III also induced a sufficient volume of the proteoglycan-rich tissue in the defect having 3.0-mm depth. Immunohistochemical staining for type-2 collagen were consistent with Safranin-O staining. G-I; a 0.5-mm gel sheet was implanted at the bottom of the defect. G-II; a 1.0-mm gel sheet was implanted at the bottom of the defect. G-III; a 5.0-mm gel plug was implanted at the bottom of the defect. G-IV; the control group has a defect left untreated. Black scale bars show a length of 1 mm.

In histological observations (Figure 4), the vacant space remained after DN gel implantation was completely filled with cartilaginous, fibrous, and/or bone tissues at 4 weeks. It is noted that the void space inside the bone indicated the presence of the DN gel, which was removed at preparation of the histological section. When the depth of the final vacant space was set at 2.0 mm, the defect was completely filled with the proteoglycan-rich tissue positive for Safranin-O staining with regenerated subchondral bone tissue in Groups I, II, and III. When the depth of the final vacant space was set at 3.0 mm, the defect was completely filled with the proteoglycan-rich tissue positive for Safranin-O staining with regenerated subchondral bone tissue in Group II and III, whereas there was little staining with Safranin-O in Group I. In the Safranin-O stained area, fairly large round cells rich in cytoplasm were scattered singly or as an isogenous group. Some specimens with hyaline cartilage regeneration appeared to be devoid of cells in the superficial layer; thereby, resembling the lamina splendens. On the other hand, the defect in Group IV was filled with the fibrous and bone tissues, independent of the depth. The edge of the adjacent cartilage showed slight reduction of Safranin-O staining without the change in cellularity. Immunohistochemical staining showed that type-2 collagen was richly expressed in the same area stained with Safranin-O (Figure 4).

### Quantitative evaluations of gross appearance and histology

**Figure 5:**
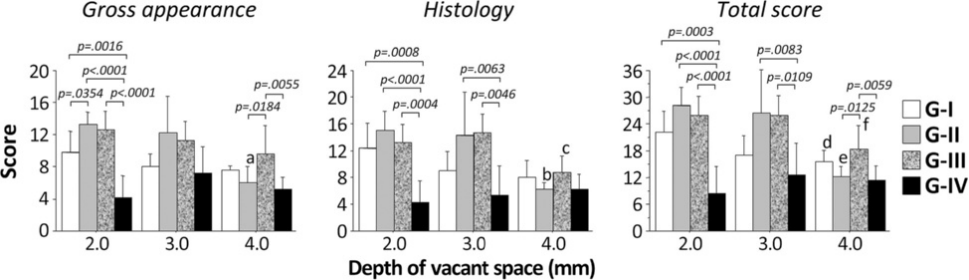
**Quantitative evaluations of gross appearance and histology according to the grading scale reported by Wayne et al.** G-I; a 0.5-mm gel sheet was implanted at the bottom of the defect. G-II; a 1.0-mm gel sheet was implanted at the bottom of the defect. G-III; a 5.0-mm gel plug was implanted at the bottom of the defect. G-IV; the control group has a defect left untreated.^a^ Significantly different from the 2.0- and 3.0-mm depths of the final vacant space in G-II (p = 0.0029 and p = 0.0074, respectively). ^b^ Significantly different from the 2.0- and 3.0-mm depths of the final vacant space in G-II (p = 0.0057 and p = 0.0100, respectively). ^c^ Significantly different from the 2.0- and 3.0-mm depths of the final vacant space in G-III (p = 0.0188 and p = 0.0038, respectively). ^d^ Significantly different from the 2.0-mm depth of the final vacant space in G-I (p = 0.0206). ^e^ Significantly different from the 2.0- and 3.0-mm depths of the final vacant space in G-II (p = 0.0032 and p = 0.0069, respectively). ^f^ Significantly different from the 2.0- and 3.0-mm depths of the final vacant space in G-III (p = 0.0299 and p = 0.0299, respectively).

Concerning the gross appearance score, the ANOVA demonstrated that there was a significant difference not only on the effect of the group (p < 0.0001) but also on the effect of the depth of the final vacant space (p = 0.0018). The post-hoc test showed that Groups I, II, and III were significantly greater than that Group IV (p = 0.0016, p < 0.0001, and p < 0.0001, respectively), and Group II was significantly greater than Group I (p = 0.0354), when the depth of the final vacant space was set at 2.0 mm. Group III was significantly greater than Groups II and IV when the depth of the final vacant space was set at 4.0 mm (p = 0.0184 and p = 0.0055, respectively). In Group II, the gross appearance score was significantly greater when the depth of the final vacant space was set at 2.0 mm and 3.0 mm than that of 4.0 mm (p = 0.0029 and p = 0.0074, respectively) (Figure 5).

Regarding the histology score, the ANOVA demonstrated a significant difference not only on the effect of the group (p < 0.0001) but also on the effect of the depth of the final vacant space (p = 0.0009). The post-hoc test showed that Groups I, II, and III were significantly greater than that Group IV (p = 0.0008, p < 0.0001, and p = 0.0004, respectively), when the depth of the final vacant space was set at 2.0 mm. Groups II, and III were significantly greater than that Group IV (p = 0.0063 and p = 0.0046, respectively), when the depth of the final vacant space was set at 3.0 mm, while there was no significant difference between Groups I and IV. There were no significant differences among the groups at 4.0 mm. In Group II, the histology score was significantly greater when the depth of the final vacant space was set at 2.0 mm and 3.0 mm than that at 4.0 mm (p = 0.0057 and p = 0.0100, respectively). In Group III, the histology score was significantly greater when the depth of the final vacant space was set at 2.0 mm and 3.0 mm than that at 4.0 mm (p = 0.0188 and p = 0.0038, respectively) (Figure 5).

Regarding the total score, the ANOVA demonstrated a significant difference not only on the effect of the group (p < 0.0001) but also on the effect of the depth of the final vacant space (p = 0.0004). The post-hoc test showed that Groups I, II, and III were significantly greater than that Group IV (p = 0.0003, p < 0.0001, and p = 0.0004, respectively), when the depth of the final vacant space was set at 2.0 mm. Groups II and III were significantly greater than that Group IV (p = 0.0083 and p = 0.0109, respectively), when the depth of the final vacant space was set at 3.0 mm, while there was no significant difference between Groups I and IV. At 4.0 mm, only Group III was significantly greater than Group IV (p = 0.0059). In Group I, the total score was significantly greater when the depth of the final vacant space was set at 2.0 mm than that at 4.0 mm (p = 0.0206). In Group II, the total score was significantly greater when the depth of the final vacant space was set at 2.0 mm and 3.0 mm than that at 4.0 mm (p = 0.0032 and p = 0.0069, respectively). In Group III, the total score was significantly greater when the depth of the final vacant space was set at 2.0 mm and 3.0 mm than that at 4.0 mm (p = 0.0299 and p = 0.0299, respectively) (Figure 5).

### Real time PCR analysis

**Figure 6:**
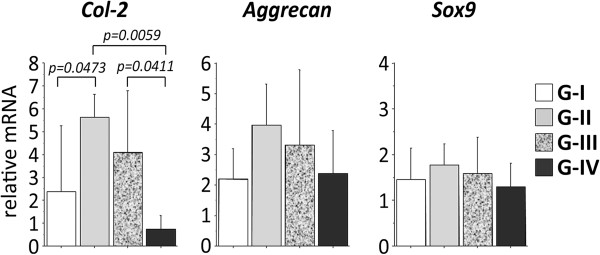
**Gene expression of type-2 collagen, aggrecan, and Sox9 in the regenerated tissue of 2-mm depth implantation at 4 weeks.** The degree of expression of type-2 collagen mRNAs was significantly greater in the regenerated tissue in G-II and III than that in G-IV.

The expression level of type-2 collagen in Groups II and III was significantly higher than that in Group IV (p = 0.0059 and p = 0.0411, respectively) and that in Group II was also significantly higher than that in Group I (p = 0.0473) (Figure 6).

## Discussion

The present study demonstrated that, when each DN gel sheet or plug was implanted at the bottom of the defect so that a 2- to 3-mm deep vacant space was left in the defect, the evaluation scores were found to be the greatest. In this implantation condition, all the evaluation scores and the expression level of the type-2 collagen gene in the two groups with implantation of a 1.0-mm thick gel sheet or a 5.0-mm thick gel plug were significantly greater than those of the untreated control group, and there were no significant differences in those parameters between the two groups. These results suggested that the *in vivo* hyaline cartilage regeneration phenomenon induced by DN gel is not influenced by the thickness of the gel implanted at the bottom of a large osteochondral defect, at least, in a range between 1.0 mm and 5.0 mm. Concerning the 0.5-mm thick sheet implantation, however, the gross observation score and expression level of type-2 collagen gene were slightly but significantly lower as compared with the 1.0-mm thick gel sheet implantation, while there were no significant differences in comparison with the 5.0-mm thick gel implantation. These results suggested that, when the thickness of the implanted gel is too thin, below 1.0 mm, the thickness of the implanted DN gel slightly but significantly reduces induction ability of the *in vivo* hyaline cartilage regeneration phenomenon.

We have studied the mechanism of the *in vivo* cartilage regeneration induced by the PAMPS/PDMAAm DN gel. Our previous *in vitro* study [20] has shown that the single-network PAMPS gel can differentiate chondrogenic ATDC5 cells into chondrocytes even in an insulin-free medium. The PAMPS network in this DN gel is negatively charged and has a sulphonic acid base, being similar to proteoglycans in normal cartilage. The PAMPS network may work as an effective reservoir of signaling molecules and growth factors like proteoglycans. This PAMPS function may create an appropriate biological environment for immature cells proliferating in the defect space, leading to chondrogenic differentiation. Another possible mechanism of the induction effect by the PAMPS/PDMAAm DN gel is related to the biomechanical condition. It is also known that mechanical microenvironment significantly affects cartilage differentiation of bone-marrow derived stem cells [21,22]. Recently, Engler et al. [23] reported that elasticity of the material on which the cultured cells are attached to directs stem cell differentiation. For example, elastic materials induce differentiation to the cartilage tissue, and stiff materials induce differentiation to the bone tissue. Therefore, the mechanical properties of the DN gel improved by the PDMAAm gel [18,19] may enhance the effect of the PAMPS gel on cartilage regeneration. In addition, we have elucidated that a dynamic physical environment is a critical factor to induce cartilage regeneration [24]. However, the mechanism of the *in vivo* cartilage regeneration induced by the PAMPS/PDMAAm DN gel has not been sufficiently clarified.

The present study has added new information in order to increase the database on the hyaline cartilage regeneration induced by the PAMPS/PDMAAm DN gel. In the present study, first, the 1.0-mm and 5.0-mm thick DN gel implants had the same ability in the *in vivo* hyaline cartilage regeneration, while the ability of the 0.5-mm thick DN gel implant was slightly but significantly inferior to that of the 1.0-mm thick DN gel implant. This fact implied that DN gel sheets thicker than 1.0 mm may provide the same physical environment under knee motion at the bottom of the defect with the 2- to 3-mm deep vacant space. Theoretically, however, when a very thin gel sheet is compressed by large forces, the apparent elastic modulus of the gel-bony floor (bone beneath the gel sheet) complex is increased by the effect of the extremely high modulus of the bony floor. Therefore, when the thickness of a DN gel sheet was 0.5 mm in this study, the apparent elastic modulus of the gel-bone complex might be too high under repetitive compression loading due to joint motion. Therefore, we speculate that the increased apparent modulus of the gel-bone complex under the repetitive compression loading may reduce the cartilage regeneration.

There are some limitations in this study. First, we used a rabbit patellofemoral joint model in this study, although this model has been commonly used to evaluate new procedures in cartilage regeneration experiments [25-27]. The joint which the most frequently requires a cartilage regeneration therapy is not the patellofemoral joint but weight-bearing regions of the femorotibial joint in the clinical field. Therefore, the results obtained cannot be simply transferred to the clinical field because different results might be obtained in the femorotibial joint model. There is a need to conduct experimental studies with a large animal model in order to fully evaluate the pre-clinical efficacy. The second limitation is that we did not actually examine the mechanical properties of the DN gel sheet or plug implanted in the bone tissue after cartilage repair occurs. The biomechanical changes of the DN gel may affect cartilage regeneration and durability because the implanted DN gel, which was seen as a void space in the histology section, was remained surrounded by bone in the defect as it was but it is unknown whether the DN gel properly works as subchondral bone with similar biomechanical properties after inducing cartilage regeneration. The third limitation is that we performed only short-term observations of the regenerated cartilage, because we intended to obtain the initial evidence that the DN gel’s thickness affected induction of the cartilage regeneration. However, we do not know the long-term effect of the DN gel on cartilage regeneration as well as the biological and biomechanical changes in the DN gel itself. To completely clarify the comprehensive effect of the DN gel thickness on the cartilage regeneration, long-term studies need to be conducted in the future. The fourth limitation is that no biomechanical evaluations of the regenerated cartilage were performed. We recognize that the biomechanical quality of cartilage is of vital importance for cartilage tissue restoration and durability. However, we believe that the biological and histological evaluations are fundamental to evaluate cartilage regeneration induced by synthetic gel implantation. The fifth limitation is that we did not evaluate the cell viability or extracellular matrix change of adjacent cartilage in the present study. As this cartilage repair method creates a large defect with a 2 to 3 mm depth of the vacant space at DN gel implantation, it might cause a significant mechanical change in the adjacent cartilage. We observed only minor influence on the adjacent cartilage in this animal model based on our previous study [16]; however, long-term studies are needed to clarify the effect of this method.

Concerning the safety of the PAMPS/PDMAAm DN gel as a biomaterial, we previously performed a pellet implantation test into the para-vertebral muscle [28], according to the guideline for biological evaluation of the safety of biomaterials, which had been published by the Ministry of Health, Labour and Welfare, Japan. Although this DN gel implantation induced a mild cell infiltration at 1 week, the degree of the inflammation significantly decreased to the same degree as that of the negative control at 4 and 6 weeks. We also cultured ATDC5 cells on the PAMPS/PDMAAm DN gel [15] as well as the single-network PAMPS and PDMAAm gels [20]. No harmful effects due to these gel surfaces were detected. We believe that the PAMPS/PDMAAm DN gel is a safe biomaterial. However, we have not completed all protocols to establish the clinical safety of this DN gel as an implant. Further studies are needed to establish the clinical safety of this gel in the near future.

As to clinical relevance, the innovative strategy with the DN gel implantation for cartilage repair without cell culture has been proposed [15,16]. We should note that this strategy is in sharp contrast to current prevailing strategies that completely fill the defect with the tissue-engineered cartilage tissue, cell-seeded scaffold material implantation, or acellular polymer scaffolds with signaling molecules [11-14]. In addition, we can expect this strategy to solve the various problems and concerns in current strategies, including donor site morbidity, two surgeries being needed, a long non-weight bearing period, a potential risk of zoonotic transmission, an enormous amount of money to establish such a tissue-engineering industry system, an extremely high medical fee for patients having the treatment, and other factors [29-36]. The results of the present study have provided important information to realize benefits of this innovative strategy with the DN gel in the near future. As described in the introduction section, a synthetic implant in the bone should be as thin and small as possible to avoid loosing the bone tissue due to the implantation. However, we do not know whether this strategy using a DN gel implantation is superior to other cartilage repair procedures such as the current autologous chondrocyte implantation technique. For the possible clinical use of this treatment strategy, further studies should be conducted to clarify these issues in the near future.

## Conclusions

The 1.0-mm thick DN gel sheet had the same ability to induce hyaline cartilage regeneration as the 5.0-mm thick DN gel plug. However, the induction ability of the 0.5-mm thick sheet was significantly lower when compared with the 1.0-mm thick gel sheet. The 1.0-mm DN gel sheet composed of PAMPS and PDMAAm is a promising device to establish a cell-free cartilage regeneration strategy that minimizes bone loss from the gel implantation.

## Competing interests

We have no financial or non- financial competing interests. We do not hold or are not currently applying for any patents relating to the content of the manuscript.

## Authors’ contribution

HM performed animal experiments and PCR analysis. NK and KY designed the study, participated in the study, and drafted the manuscript. NK, KA, and FK participated in designing the study and instructed animal experiments. TK and JPG created the DN-gel material. All authors read and approved the final manuscript.

## Pre-publication history

The pre-publication history for this paper can be accessed here:

http://www.biomedcentral.com/1471-2474/14/50/prepub
